# Biotea-2-Bioschemas, facilitating structured markup for semantically annotated scholarly publications

**DOI:** 10.5808/GI.2019.17.2.e14

**Published:** 2019-06-19

**Authors:** Leyla Garcia, Olga Giraldo, Alexander Garcia, Dietrich Rebholz-Schuhmann

**Affiliations:** 1 EMBL-EBI, Wellcome Genome Campus, Hinxton, CB10 1SD, UK; 2 ELIXIR Hub, Wellcome Genome Campus, Hinxton, CB10 1SD, UK; 3 Ontology Engineering Group, Campus de Montegancedo, Boadilla del Monte, Universidad Politécnica de Madrid, Madrid, Spain; 4 BASF SE, G-FDR/BI-G200, 67056 Ludwigshafen am Rhein, Germany; 5 ZB MED-Information Centre for Life Sciences, 50931 Köln, Germany

**Keywords:** biomedical text mining, literature metadata, semantic annotations, structured data, web page markup

## Abstract

The total number of scholarly publications grows day by day, making it necessary to explore and use simple yet effective ways to expose their metadata. Schema.org supports adding structured metadata to web pages via markup, making it easier for data providers but also for search engines to provide the right search results. Bioschemas is based on the standards of schema.org, providing new types, properties and guidelines for metadata, i.e., providing metadata profiles tailored to the Life Sciences domain. Here we present our proposed contribution to Bioschemas (from the project “Biotea”), which supports metadata contributions for scholarly publications via profiles and web components. Biotea comprises a semantic model to represent publications together with annotated elements recognized from the scientific text; our Biotea model has been mapped to schema.org following Bioschemas standards.

**Availability:** Data and software used are available at https://doi.org/10.5281/zenodo.2595281.

## Introduction

The high and sustained growth rate in scholarly publications requires ever more efficient ways to identify the most relevant documents for the own research work and increasingly, the scientific text is exploited to guide this search. This requires Named Entity Recognition and Text-Mining approaches, but also effective ways to expose identified data as structured metadata and to interlink to data and metadata with external sources such as ontologies and fact repositories, which is still a challenging research domain. The Journal Article Tag Suite (JATS) serves as the preferred (semi)structured approach for publishers to provide machine-readable access to scholarly articles [1]. Although XML is a well-established and shared format, it falls behind in the efficient use of semantics, where Resource Data Framework (RDF)/XML or JSON-LD deliver better results. However, deploying a RDF infrastructure approach can only be achieved with significant overheads in comparison to offering RESTful services for the reason that data (and metadata) is more readily available through RESTful services in contrast to data provision through XML repositories. Schema.org forms a lightweight alternative, i.e., allows semantic annotation at low development overheads, in comparison to complete semantic environments based on ontologies and RDF approaches.

Schema.org is a collaborative initiative offering a simple yet effective way to add structured metadata to web pages via markup: making pages more findable and improving search results as search engines can identify better the clues what a page is about. Schema.org also eases the ways to interlink related resources; for instance, a movie can be linked to its actors, directors or similar movies. Such interlinking of web resources can boost interoperability, allowing search engines to move from mainly presenting search only to providing comprehensive summaries. Scholarly literature repositories such as Zenodo and EuropePMC have recognized the potential of schema.org and currently support markup on their pages. However, they mostly limit to metadata, i.e., authors, title, abstract and journal, leaving aside any entity of interest from the life science domain that could be recognized from the text. Despite its popularity, the high diversity of properties in schema.org makes it still difficult for not well prepared data scientists to adopt such markup. Bioschemas, a community and collaborative project, focuses on integrating Life Science types to schema.org and, at the same time, makes its adoption easier via profiles tailored to the Life Science domain [2]. A profile comprises a fixed combination (a “set”) of guidelines regarding minimum, recommended and optional properties, well-known ontology terms to be used together with the properties and examples, so that users can use them as a template to markup their own resources.

Biotea proposes a conceptual model to represent scholarly publications as Linked Data [3,4], covering not only the metadata but also the article structure, content and semantic annotations, where a semantic annotation corresponds to a named entity recognized from the text. Biotea currently supports annotations obtained from the National Center for Biomedical Ontology (NCBO) annotator service [5]; however, additional annotators could be added. In order to facilitate the adoption of schemas.org markup for scholarly articles as well as to add support to semantic annotations, Biotea has joined the Bioschemas effort by adding five scholarly publication related profiles. Here we report on the Biotea draft profiles proposed to Bioschemas and on two web components capable to render schema.org markup for scholarly articles metadata together with annotations, using the PubMed Central Open Access subset (PMC-OA) as main input. As a result, we anticipate a knowledge graph derived from the scientific literature and based on Bioschema. This type of graph eases the way towards co-citation networks, author-expertise networks, and ontology-based associations among others.

## Biotea Draft Profiles for Bioschemas

Biotea profiles proposed to Bioschemas (as a draft) include journal, volume, issue, scholarly article and semantic scholarly annotation (http://bioschemas.org/groups/Biotea2Bioschemas). These profiles corresponding to journal, volume, issue and scholarly article are reasonably stable as they correspond to regular metadata and data that have been well-established for publications, e.g., title, authors, abstract, journal, publisher, pages, dates and citations. Biotea supports two ways to represent annotations, one based on the Annotation Ontology [6] and another one on the Open Annotation Ontology [7]. None of them can be fully mapped to schema.org, therefore modifications are more likely to be introduced to our SemanticAnnotation profiles, so it can adapt well to a variety of annotators and annotated documents. Currently we are using such profiles for annotations recognized in scholarly documents, but it could as well be deployed, for instance, to annotate chemicals in patent documents. As more use cases will be explored, further adjustments will be needed and should be addressed before moving from a draft to a supported profile.

**Fig. 1.**
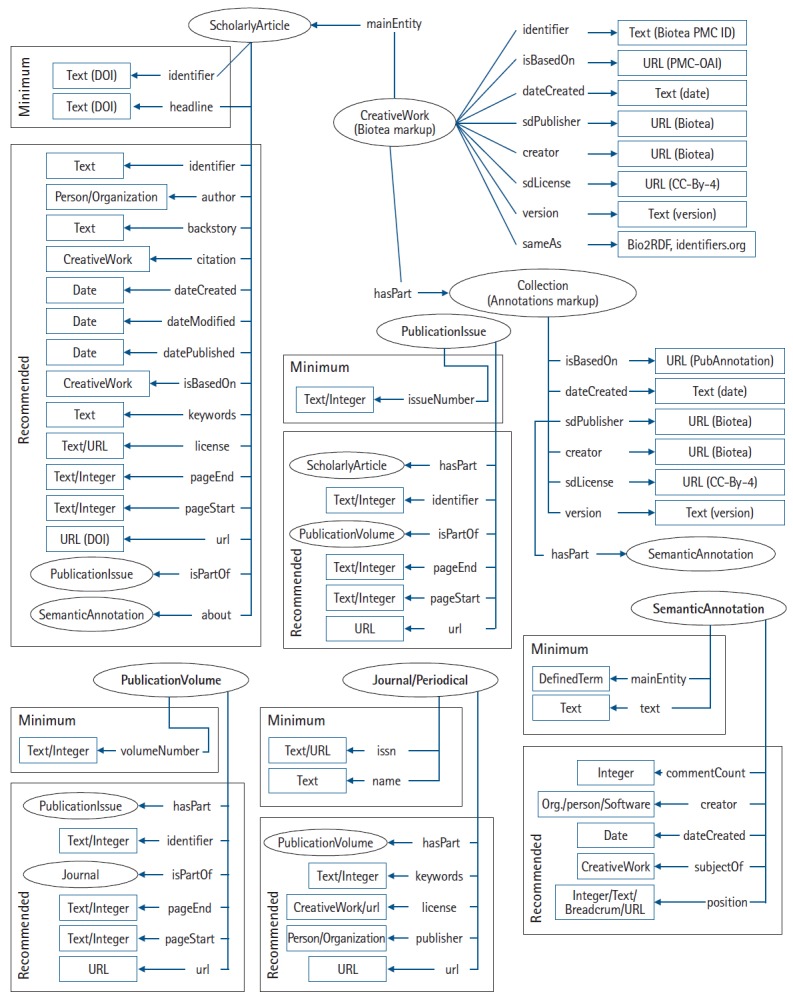
Biotea model mapped to schema.org. Following Bioschemas approach, minimum, recommended, and optional properties are proposed (only the former two included in the diagram).

The publication is represented as a schema:ScholarlyArticle part of a periodical publication, i.e., a journal with possibly a volume and issue. The property schema:about is used to link the publication to its annotations which link back to the article via schema:subjectOf. An overview of our Biotea mapping to Bioschemas is shown in Fig. 1.

One of the key aspects in Biotea’s mapping to schema.org is the separation between the structured data and the publication itself. Schema.org includes some properties in schema:CreativeWork, that should be used only for a structured data representation. However, those properties are still limited, i.e., do not cover all the needs regarding structured metadata on top of scholarly publications. For instance, structure metadata can be split into different parts, e.g., linksets, but there is only one schema:hasPart property for schema:CreativeWork. In order to allow for more input, we therefore keep the structured data model separated from the publication, the former modelled as a schema:CreativeWork and the latter as a schema:ScholarlyPublication; the structured data is linked to the publication via schema:mainEntity. Through this separation in combination with the use of a lightweight semantic approach such as schemas.org, we aim to improve our approaches for FAIRability for publications, since this type of data gives better compliance (in comparison to JATS/XML) with the Findability, Accessibility, Interoperability and Reusability (FAIR) principles [8].

## Biotea-2-Bioschemas Web Components

Web components make it easy to add customized elements to web pages so they can easily be embedded into any web page. In order to parse and render scholarly publication metadata as well as semantic annotations from their text, we have created two web components. Our web components take their input, parse it and render it following the corresponding Bioschemas markup. The markup is added at the end of the web page as a new element, such element corresponds to a script with the MIME-type “application-json”. We have chosen this asynchronous approach as it makes it easier for web page providers to optimize the end-user content, leaving any Biotea-Bioschemas machine-readable bits to the end. Rendering the markup as JSON-LD also provides readable HTML, as it keeps the content separated from the markup. A possible drawback in this approach is the uncertainty regarding the capacity of crawlers to support asynchronous loading of the structured metadata. Asynchronous loading is nowadays a common approach on web page development and therefore should be supported by main search engines when it comes to structured metadata. Bioschemas has already recognized this and therefore has initiated efforts to contribute with a crawler capable to deal with delayed loading (https://github.com/ricardoaat/bioschemas-gocrawlit).

The Biotea-bioschemas-metadata web component requires as input a JATS XML response from the PMC-OA Interface service (PMC-OAI, https://www.ncbi.nlm.nih.gov/pmc/tools/oai/). It converts such a response into a JSON object and then selects elements of interest such as title authors, publication data and abstract and map them to the Biotea-Bioschemas profiles. For our second web component, biotea-bioschemas-annotations, we use PubAnnotation [9] annotations as main input. Although it is possible to use the NCBO annotator service on the fly, doing so poses a problem regarding the obtained annotations. The NCBO annotator works with the current version of the ontologies hosted by the NCBO ontology portal (https://bioportal.bioontology.org/), as ontologies are updated, so could annotations using such ontologies, meaning that a different set of annotations for the same article could be recovered at different times. In order to avoid this, we have created a Biotea project in PubAnnotation so we can host there a set of frozen annotations used to provide the corresponding Bioschemas markup.

**Fig. 2.**
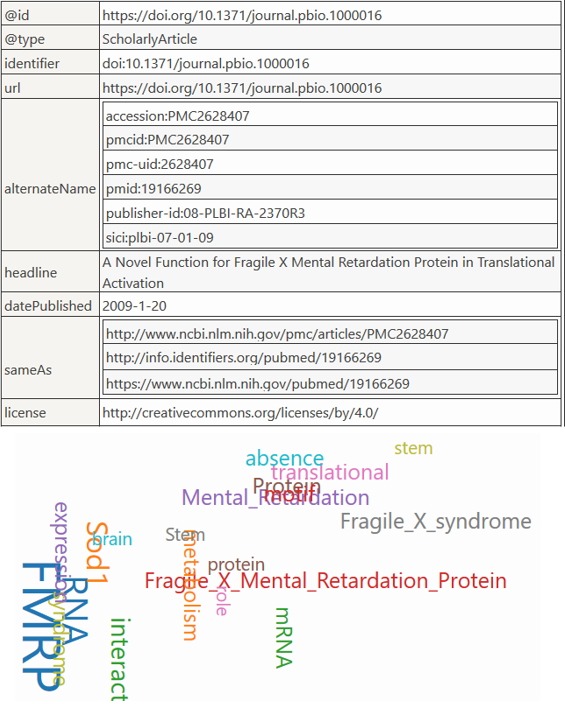
Overview of the metadata markup showed as a table, annotations with more than 1 occurrence are shown as a cloud of words.

As a proof of concept, we have uploaded 2,596 full text articles, annotated their abstracts with 13 ontologies from Bioportal, covering gene, protein, drug, disease and symptom areas, for a total of 894,926 annotations, and exposed them via PubAnnotation. Our web components can be seen in action in our Biotea-Bioschemas main page (http://biotea.github.io/bioschemas). Although it is possible to use our web components with any PMC-OA article or any PubAnnotation PMC hosted annotation set, we have limited our showcase to those 2,596 articles currently hosted at PubAnnotation. All of the articles are included in a sitemap, making it easier for search engines to index the content. In Fig. 2 we show the markup corresponding to PMC-OA article identified as PMC2628047, the corresponding URL is http://biotea.github.io/bioschemas?pmc=2628047.

## Conclusions and Future Work

By mapping Biotea to schema.org following the standards proposed by Bioschemas, we are preparing the basis towards literature-based knowledge graphs based on Bioschemas. At this point, it remains open what types of inferences based on such a graph could be drawn, under the consideration that the semantics given by schema.org is less strict in comparison to ontology-based approaches such as https://scigraph.springernature.com. Schema.org and Bioschemas are, however, easier to adopt as adding markup to HTML does not require such a robust infrastructure as ontology-based approaches do. As a future work, our initial step on realizing a Bioschemas knowledge graph potential would be extracting author-expertise networks. From there, we hope more developments will come, exploring more complex associations such as target-disease.
